# Hyperprogressive disease in non-small cell lung cancer treated with immune checkpoint inhibitor therapy, fact or myth?

**DOI:** 10.3389/fonc.2022.996554

**Published:** 2022-11-29

**Authors:** Alec S. Britt, Caitlyn Huang, Chao H. Huang

**Affiliations:** ^1^ University of Kansas Comprehensive Cancer Center, Westwood, KS, United States; ^2^ Pembroke Hill High School, Kansas City, MO, United States; ^3^ Kansas City VA Medical Center, Kansas City, MO, United States

**Keywords:** checkpoint inhibition therapy, hyperprogression, non-small cell lung cancer, definition, mechanism

## Abstract

The therapeutic landscape for patients with non-small cell lung cancer (NSCLC) has dramatically evolved with the development and adoption of immune checkpoint inhibitors (ICI) as front-line therapy. These novel antibodies target the interactions in immunoregulatory pathways, between programmed death-1 (PD-1) and programmed death ligand-1 (PD-L1), or cytotoxic T-lymphocyte antigen 4 (CTLA-4) and B7, resulting in the activation of T cells and cytotoxic response to induce an immunologic response. ICIs have demonstrated significant survival benefits and sustained responses in the treatment of NSCLC leading to the long-term survival of up to 5 year. One unusual response to ICI is a phenomenon termed Hyperprogressive Disease (HYD), which occurs in a subset of patients for whom ICI therapy can induce rapid disease growth, which ultimately leads to poorer outcomes with an incidence rate ranging from 5 to 37% in NSCLC patients. Prior reviews demonstrated that HYD can be defined by rapid tumor progression, deterioration of patient’s symptoms or new onset of disease. The mechanism of HYD could be related to genomic and tumor microenvironment changes and altered immune response. It will be important to establish a common definition of HYD for future research and clinical care.

## Introduction

The therapeutic landscape for patients with non-small cell lung cancer (NSCLC) has dramatically evolved in the last several years with the development and adoption of immune checkpoint inhibitors (ICI) as front-line therapy. These novel antibodies target the interactions in immunoregulatory pathways, such as those between programmed death-1 (PD-1) and programmed death ligand-1 (PD-L1), or cytotoxic T-lymphocyte antigen 4 (CTLA-4) and B7, resulting in the activation of T cells and cytotoxic response to induce an immunologic response in several solid tumor types (1). ICIs have demonstrated significant survival benefits and sustained responses in the treatment of NSCLC leading to the long-term survival of up to 5 years (2–4).

One unusual response to ICI is a phenomenon termed Hyperprogressive Disease (HYD), which occurs in a subset of patients for whom ICI therapy can induce rapid disease growth, which ultimately leads to poorer outcomes (5). Existing data suggest an incidence rate ranging from 5 to 37% in NSCLC patients (6–8). Despite this known entity, a consensus definition for the diagnosis of HYD has not been determined, and the explication of underlying pathophysiologic mechanisms has remained elusive. In this review, we will evaluate recent data on HYD in the NSCLC population, as well as discuss the proposed mechanisms, predictors, and biomarkers potentially implicated in the process.

## Case illustration

**Figure 1:**
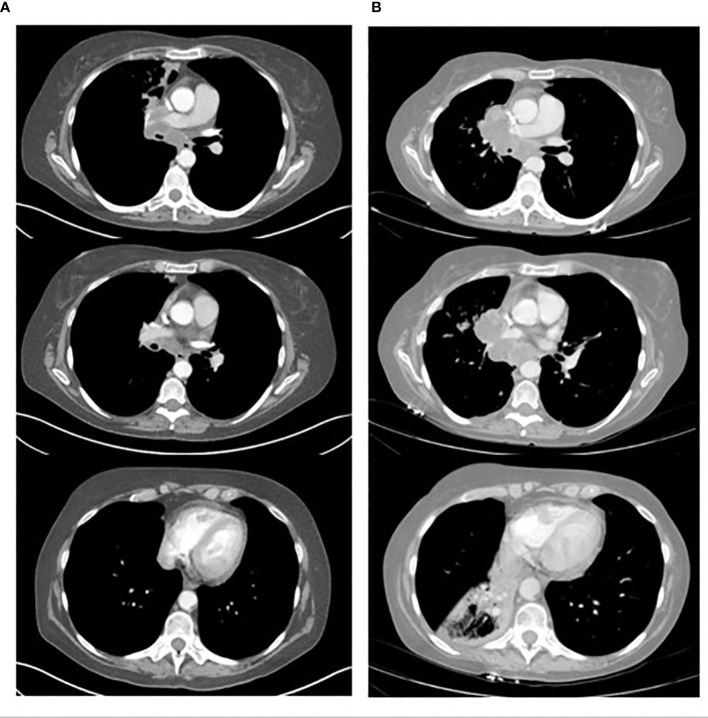
Shows the CT scans of a 61-year-old patient with NSCLC. **(A)** shows 3 axial CT scan images of the tumor located in the right para-mediastinal area and lymph node enlargement at level 7 and 10. **(B)** corresponding axial CT scan images after 2 cycles of Pembrolizumab monotheraphy (49 days later) showing clear increase in tumor mass and lymph nodes at level 7 and 10 and atelectasis of right lower lobe.

61-year-old white female presented on 3/17/2022 with several months of cognitive changes (including confusion) and gait instability. CT of the head done on 3/17/2022 showed a large frontal lobe mass measuring 3.2 x 2.6 cm with extensive adjacent edema of the left frontal and parietal lobes, midline shift of 7 mm, and marked compression/distortion of the left frontal horn of the left lateral ventricle. The patient was admitted to the neurosurgery service and CT chest/abdomen/pelvis on 3/18/2022 showed a 2.1 cm nodule in the medial azygos lobe of the right upper lobe, compatible with primary lung ca. There were several smaller irregular ground glass and nodular opacities in the left lower lobe (indeterminate or synchronous malignancies or metastases). She also had mild mediastinal and right hilar lymphadenopathy, but no abdominal pelvic metastatic disease. The patient underwent endobronchial ultrasound biopsy and bronchioalveolar lavage on 3/21/2022. Fine Needle Aspiration of the right hilar mass showed poorly differentiated carcinoma pulmonary non-small cell carcinoma. PD-L1 TPS was 40%. MRI of the brain done on 3/22/2022 showed enhancing anteromedial left frontal cerebral cortical nodule, indicating solitary cerebral metastasis, marked associated left anterior cerebral vasogenic edema, and mild rightward frontal midline shift. The patient underwent a left frontal craniotomy on 3/23/2022 and pathology showed poorly differentiated carcinoma consistent with metastasis from lung adenocarcinoma primary. PD-L1 TPS was 1 to 2%. NGS testing showed mutation of TP53, BRAF L597Q (not V660E), STK-11, PDGFRA, KMT2D, ZNF217, and RNA testing was negative for an actionable mutation. She was discharged on 3/25/22 and underwent post-operative radiation to the surgical bed from 4/12/22 to 4/25/22. She tapered off Decadron and enrolled in a trial randomized to Pembrolizumab single agent which started on 5/13/22. After 2 cycles of therapy, the patient developed deterioration of her performance status, and required hospital admission 6/30/22. The CT scan after 2 cycles done on 6/23/22 showed significant progression of her disease (**Figure 1**).

## The definition and diagnosis of HYD

HYD is generally described as unexpected, accelerated tumor growth after treatment with ICI therapy (9). Early after governing approval and real-world application of ICI in therapies for solid tumor patients, this phenomenon was often reported anecdotally as a disease flare, with an increased size of cancer lesions noted on imaging noted shortly after initiation of such treatment (10). Although this can be attributed to the natural progression of the disease, the course of HYD is punctuated by the unproportioned growth of the disease compared to the course of the disease before therapy and significant deterioration of the patient’s condition. It should be noted that while the literature on HYD more recently has been centered around single-agent ICI therapy. The shape of progression-free survival curve in Checkmate 277 and Mystical trial, suggests that this also occurs when using dual ICI (anti-PD-1 with anti-CTL4 combination) (11, 12). HYD has been described in chemotherapy, in about 5% (6), and tyrosine kinase inhibitor therapies, varying up to 25% (13), as well. However, these studies are not definitive that HYD exists in non-immunotherapy treated patients since both the population and definition criteria were very heterogeneous (5, 6).

Despite increased recognition of the hyperprogressive phenomenon, there is a lack of a unifying definition of this process. Several previous studies have sought to define HYD across a broad range of tumor types. Definitions thus far largely have been categorized into time-dependent criteria or size/clinically dependent criteria.

Examples of time-dependent criteria employed in the literature include tumor growth rate (TGR) and tumor growth kinetics (TGK). TGR calculation involves the difference (or ratio) of 3-dimensional tumor volume per month, related to the sum of the target lesion(s) diameter(s) as well as the time between imaging evaluations (14). Tumor growth kinetics (TGK) on the other hand is a function of 2-dimensional tumor diameter over time.

Champiat et al. (2017) were the first to collectively describe and define HYD in solid-tumor patients who were treated with immunotherapy (5). This retrospective analysis of 131 patients evaluated the prevalence of hyperproliferative disease in those treated in phase I clinical trials with immunotherapy. The authors defined the HYD as progression at first evaluation with a TGR ratio increase of two-fold or higher by RECIST 1.1 criteria. Of note, this study included only 13 patients with lung cancer, none of whom developed HYD (5, 6).

A later study by Singavi et al. incorporated a similar definition to the criteria set out by Champiat, including a TGR increase of two-fold or higher per RECIST 1.1 criteria, with an additional requirement of RECIST 1.1 tumor size increase of 50% or higher (15). Eventually, data from Ferrara et al. in 2018 would evaluate HYD in NSCLC patients using a definition of progression per RECIST 1.1 criteria as well as TGR difference (rather than a ratio) of 50% or higher (6).

Additional data from Kato et al. (2017) reviewed 155 patients with advanced solid tumors who received immunotherapies and had their tumors evaluated by next-generation sequencing. This study defined HYD as a TTF of fewer than 2 months, a 50% or higher increase in tumor burden compared to pretreatment imaging, and a 2-fold or higher increase in progression pace (16). Notably, this study had a total of 38 NSCLC patients included, with 18 of these patients experiencing a TTF in less than 2 months. Saâda-Bouzid et al. (2017) evaluated HYD in 34 patients with squamous cell carcinoma of the head and neck, with the definition of HYD relying on TGK (17, 18).

The heterogeneity in definitions of HYD has real-world implications in the current diagnosis of HYD. A recent retrospective cohort study evaluated 406 patients with NSCLC, analyzing the incidence and outcomes in a single population of patients with HYD as defined by five different, established definitions per previous trials. The data revealed a variance in reported incidence (5.4%-18.5%) of HYD, with concordance between definitions ranging from 33.3% to 69.3% (19). Indeed, a previous meta-analysis and systematic review of 3109 patients across 24 studies suggest that despite being a distinct outcome, the lack of a standardized, validated definition of HYD leads to significant variability in reported incidence (20). Given the implications of HYD on survival outcomes, it is of great interest to oncologic physicians to standardize definitions of this phenomenon in the future.

Beyond HYD, other patterns of progression on ICI have been described in the literature. Gandara et al. described fast progression (FP) and early death (ED) in a retrospective evaluation of the OAK study. FP was defined on size-based criteria (50% or greater increase in the sum of largest diameters of target lesions per RECIST 1.1 criteria) and did not require pre-baseline assessment. ED was defined as death due to disease progression within 12 weeks from baseline in patients without a response assessment (21). Further evaluation has suggested that these are distinct patterns of progression with limited overlap between the groups (22).

The use of parameters such as the TGR or TGK allows for the evaluation of tumor kinetics as guided by tumor size. Of note, the TGK does not involve a three-dimensional evaluation of tumor size, which may lead to some overestimation of the incidence of HYD (5, 6, 15, 18). These time-based criteria require at least three radiologic examinations (pre-baseline, baseline, and post-treatment) to allow for a dynamic assessment of tumor growth momentum (5). This allows for differentiation of the natural course of the disease (in which tumor growth curves would largely remain similar before and after treatment) versus true HYD, in which tumor growth speed would increase after initiation of ICI. Unfortunately, time-dependent criteria cannot be readily applied to all patients in a first-line setting, as often these patients do not have pre-baseline imaging.

Size or clinically dependent criteria require pre-baseline imaging but do require dynamic data regarding tumor momentum in growth, i.e., RECIST criteria measuring size (13) or reliant on the changes in the patient’s clinical condition (23). Another criterion is time to treatment failure (TTF), defined as the time from the start of treatment with ICI to its discontinuation, increase in the sum of target lesions from baseline imaging to current radiologic evaluation, the appearance of new lesions from baseline imaging, or clinical deterioration.

Matos et al. used RECIST and defined HYD as a progression of disease within the first 8 weeks after treatment with ICI, an increase of a minimum of 10mm and addition to increasing≥ 40% in the sum of target lesions compared with baseline (double of the RECIST 1.1 definition of progression) and/or increase of ≥ 20% in the sum of target lesions compared with baseline and the appearance of new lesions in at least 2 different organs. In this study, they analyzed 287 patients treated with ICI monotherapy or in combination. HYD by RECIST definition occurred in 10.7% of patients representing 27.1% of patients with disease progression. Their outcome was worse with median overall survival (mOS) of 5.23 months vs. 7.3 months without HYD (13).

Furthermore, size- or clinical-dependent criteria may be easier to implement in the real-world setting and possibly in clinical trials. However, these evaluations cannot describe the rates or speed of tumor growth inherently associated with time-based evaluations, and thus distinguishing between natural disease progression and HYD remains difficult (24). A limitation of size-dependent criteria like RECIST could be potentially overestimating HYD when the disease has rapid TGR, but even with this limitation patients with rapid TGR are also likely to have a worse outcome and are of clinical significance (13).

Future implementation of early disease assessments and integrating time-based tumor kinetic evaluation will be crucial in identifying those with HYD. A proposed set of parameters as the definition of HYD based on the review of the literature is shown in **Table 1**.

**Table 1:** Proposed criteria of Hyperprogressive disease.

**Tumor measurement criteria**
1- Increase of two-fold or higher per RECIST 1.1 criteria OR 50% or higher increase in tumor burden compared to pretreatment imaging
2- Time to progression less than 3 cycles of therapy (2 months)
3- 2-fold or higher increase in progression pace
4- Progression of new lesions
**Patient symptoms criteria**
5- Rapid decrease in baseline performance status or worsening of symptoms related to the disease progression
6- New onset of complications related to disease progression i.e. SVC, increase pleural effusion
**Laboratory Criteria**
7- **LDH > upper limit of normal**
**Measurement Methods**
Tumor Growth Rate (TGR) 3 D difference in tumor volume per month, related to the sum of the target lesion(s) diameter(s) as well as the time between imaging evaluations.
Tumor Growth Kinetics (TGK) 2D as a function of tumor diameter over time.
**Potential Factors associated with HYD**
** 1. Increased Age**
** 2. Higher tumor burden with 2 or more metastatic sites with one of the liver**
** 3. High LDH**

## Proposed mechanisms of HYD

While the process of HYD in NSCLC with ICI therapy has been increasingly documented, the mechanisms responsible remain relatively unknown. Several proposed hypotheses and mechanisms have been suggested, including factors involving expansion of PD-1 expression and T regulatory cell, changes in the immunosuppressive tumor microenvironment, the diminished response of anti-tumor immune cells to ICIs, and the involvement of alternative signaling networks *via* oncogenic driver mutations (25). A summary of the proposed mechanisms is shown in **Table 2**.

**Table 2:** Mechanism of hyperprogression.

Genomic changes	Changes in tumor microenvironment	Altered immune response
MDM/MD4 (↑ VEGF) Deletion Mutations JAK-STAT activation EGFR activation DNMT3A mutation ↓ Antigen processing genes	↑ VEGF ↑ M2 Microphage ↑ IFN-γ ↑T-Reg	Activation of Fcy receptor Change Ratio of Effector/Regulatory T cells ↑PD-1 expression Activation of alternative inhibitory receptors: TIM3, TIGIT, and LAG3 Immunesenescense: T cells (CD28-CD57+KLRG1+).
Resulting effect		
Abnormal signaling	Immunosuppression	↓ Anti-tumor response to ICI

ICI, Immune-checkpoint inhibitors; VEGF, Vascular Endothelial Growth Factor; EGFR, Epidermal Growth Factor Receptor; DNMTA3A, DNA methyltransferase 3A T-cell; TIM3, T-cell immunoglobulin and mucin domain-containing protein 3; TIGIT, T cell immunoglobulin and ITIM domain; LAG3, Lymphocyte activation gene-3; KLRG1, killer-cell lectin like receptor G1. ↑, Increase; ↓, Decrease.

It has been suggested that the use of ICI can lead to the expansion of regulatory T cells, which are immunosuppressive cells that may proliferate in the setting of PD-1 or PD-L1 blockade. A study by Kamada et al. showed that patients without HYD showed a markedly decreased ratio of regulatory T cells to CD8^+^ T cells, whereas those with HYD showed no significant change to maybe a slight increase ratio of regulatory T cells (26). This may lead to increased immunosuppression and tumor hyperprogression. T cell exhaustion, or T cell dysfunction, may also be implicated in ICI therapy, possibly as a result of upregulation of alternate inhibitory receptors such as T-cell immunoglobulin and mucin domain-containing protein 3 (TIM3), T cell immunoglobulin and ITIM domain (TIGIT), and Lymphocyte activation gene-3 (LAG3) (27, 28). Additionally, highly differentiated, circulating senescent T cells may have implications in the role of HYD, as it has been identified that those with HYD NSCLC (and those that did not respond to anti-PD1/PD-L1 therapy) have an increase in this T cell population after ICI therapy (29). Recent data have further supported the hypothesis that circulating T cell immunosenescence plays a role in ICI responsiveness. Ferrara et al. reported that 28% of 83 advanced non-small cell lung cancer patients were observed to have circulating senescent T cells. Among them, 4 patients had HYD with a delta of TGR>50 and all of them had between 47% to 63% of circulating CD8 T cells with a senescent immunophenotype (CD28- CD57+ killer-cell lectin-like receptor G1 (KLRG1+)). None of them had a response compared to 30% in patients without T cell immunosenescence markers (30).

The tumor microenvironment plays a significant role in responses to ICI therapy, and it has been proposed as a potential mechanism in the development of HYD as well. ICI-induced upregulation of immunosuppressive cytokines, including interleukin 10 and interferon-gamma (IFN-γ), may lead to IFN-γ-dependent recruitment of immunosuppressive myeloid-derived suppressor cells (31). Inflammatory cell presence in the tumor microenvironment can lead to tumor escape from ICI in a variety of mechanisms including local inflammation, modifying metabolism, and increased angiogenesis. A study by Lo Russo et al. analyzed 152 patients with NSCLC who underwent treatment with immunotherapy, and in patients with HYD there was an increased population of tumor-associated macrophages, and it has been theorized that this relationship may be due to increased interaction between the macrophages and the Fc fragment of the ICI antibodies (23).

Specific genomic mutations have also been posited as driver events for HYD. The study by Kato et al. (16) noted an association between HYD and MDM2/MDM4 amplification. This may be related to dysregulation of p53 and resultant downstream Vascular Endothelial Growth Factor (VEGF) upregulation, as MDM2 directly leads to p53 degradation *via* proteasome (32). ICI therapy leads to increased JAK-STAT signaling, with a resultant increase in interferon-regulatory factor (IRF)-8 expression, leading to downstream MDM2 expression (33). Epidermal Growth Factor Receptor (EGFR) activation is also associated with the upregulation of tumor immune escape markers (PD-1/PD-L1, CTLA4), and is associated with a slight increase in the risk of developing HYD (15, 16, 34).

## Predictive features and outcomes of HYD

With the accelerated tumor growth noted in this subset of patients, a focus on potential predictive factors has been highlighted in previous data. These data sets span several different solid tumor subtypes, but more recent studies have highlighted specific risk factors in the NSCLC patient population.

The association between HYD and age is not entirely clear. Several studies have shown that patients who are older when treated with ICI have a higher risk of developing HYD (5, 35). This could be due to noted declines in T cell immunity as patients age (36). However, other studies have not shown an association between HYD and age. In the 2018 data from Ferrara et al, the first study to specifically address HYD in an NSCLC population, this association with age was not seen, although the definition of hyperprogression did differ (6).

Some studies have found a correlation between metastatic burden, locoregional recurrence, and risk of HYD. Head and neck cancer patients in one study were found to have a higher incidence of hyperprogression in those with metastatic cervical nodes versus those without, as well as a higher rate of regional recurrence noted in those who had developed HYD (18). In NSCLC patients, those with a higher metastatic burden at the time of treatment were more likely to develop HYD, although the mechanism behind this is unclear (6).

As previous data have indicated, amplification of MDM2 and alterations of EGFR are associated with an increased risk of HYD. NGS evaluation of patients with hyperprogression revealed MDM2/MDM4 amplification in 6 different patients (16). Additional data support the association between copy number alterations in MDM2/MDM4, as well as EGFR and several chromosome 11 alterations, and HYD (15). The study by Kato et al. also noted DNA methyltransferase 3A (DNMT3A) alterations as an independent predictor of poorer clinical outcomes with ICI therapy (16). Additionally, previous studies seem to suggest a possible role for other markers such as lactate dehydrogenase (LDH) and derived neutrophil to lymphocyte ratio (37), although this has not been reliably replicated in all studies.

While previous data has largely included multiple solid tumor subtypes in the analysis of hyperprogression, more data specific to NSCLC patients has been elucidated. A recent systematic review and meta-analysis compared 6 studies with 1389 NSCLC patients and identified five different factors significantly associated with the risk of HYD, including an Eastern Cooperative Oncology Group (ECOG) score greater than 1, Royal Marsden Hospital (RMH) score of two or higher, serum LDH greater than the upper limit of normal, more than two metastatic sites, and presence of liver metastasis (38). Ferrara et al. demonstrated, as previously stated, an increased risk of HYD in patients with a higher number of metastatic sites, but no correlation between age, LDH, neutrophil to lymphocyte ratio, or MDM2 or EGFR mutations (6).

The development of HYD is largely associated with a poorer prognosis in the available literature. Early data from Champiat et al. revealed an mOS of 4.6 months in patients with hyperprogression (vs. mOS of 7.6 months in those without), with another study by Kim et al. showing an mOS of 50 days in patients with HYD (vs. 205 days in those without) (5, 8). In the NSCLC-specific population in the data by Ferrara et al, HYD was associated with a particularly poor survival if it developed within the first 6 weeks after starting ICI therapy (3.4 months vs 6.2 months) (6).

## Conclusion

Since the advent and adoption of ICI therapy in the treatment of advanced NSCLC, multiple studies have shown significant improvements in outcomes for these patients (3, 4), but occasionally patients can develop a paradoxical rapid acceleration of tumor growth labeled as HYP. HYP remains a challenge in patient management for the oncology physician due to variable definition, lack of an easily measurable biomarker, and HYD’s implications for therapeutic choice and outcomes for patients. The debate about which criteria should be adopted among time-dependent or size-related variables is ongoing. A selected combination of these criteria may be used in a universal definition of HYD in the future. Further research into the mechanism of HYD in T cell regulation, changes in the tumor microenvironment, and genomic changes could eventually lead to the identification of a potential biomarker of HYD. This could complement subjective criteria like clinical parameters and settle cases that are in doubt. While the body of literature is increasing, there is a relative dearth of high-quality data related to hyperprogression, as the majority of studies are limited to retrospective reviews. Therefore, the development of universal HYD definition criteria and identification of a reliable biomarker will be paramount to establish HYD as a formal entity recognized by academic oncologists and governing agencies and allow for uniform diagnosis to be applied in prospective clinical trials. This will spur the design of therapeutic investigations that will guide the future management of HYD and change the trajectory of HYD in the field of immune-oncology.

## Author contributions

CHH review and editing of manuscript, conceptualization of the review, tables and figures, and literature review. AB literature review, draft of manuscript and literature review. CH review and editing, creation of figures. All authors contributed to the article and approved the submitted version.

## Funding

Professional CME funds.

## Conflict of interest

CHH is an investigator in clinical trial funded by Genetech, BMS, Amgen, RRX, Pfizer, Exelixis, Incyte, Sanofi and Mirati.

The remaining authors declare that the research was conducted in the absence of any commercial or financial relationships that could be construed as a potential conflict of interest.

## Publisher’s note

All claims expressed in this article are solely those of the authors and do not necessarily represent those of their affiliated organizations, or those of the publisher, the editors and the reviewers. Any product that may be evaluated in this article, or claim that may be made by its manufacturer, is not guaranteed or endorsed by the publisher.
